# Time below range alone is insufficient to identify severe hypoglycaemia risk in type 1 diabetes—the critical role of hypoglycaemia awareness: results from the SFDT1 study

**DOI:** 10.1007/s00125-025-06536-x

**Published:** 2025-09-09

**Authors:** Dulce Canha, Pratik Choudhary, Emmanuel Cosson, Isabela Banu, Sara Barraud, René Valéro, Nathalie Ronci, Blandine Delenne, Lise Dufaitre, Tiphaine Vidal-Trecan, Pauline Schaepelynck, Caroline Sanz, Sopio Tatulashvili, Gloria A. Aguayo, Guy Fagherazzi, Jean-Pierre Riveline

**Affiliations:** 1 Deep Digital Phenotyping Research Unit, Department of Precision Health, Luxembourg Institute of Health, Strassen, Luxembourg; 2 Faculty of Science, Technology and Medicine, University of Luxembourg, Esch-sur-Alzette, Luxembourg; 3 Diabetes Research Centre, University of Leicester, Leicester, UK; 4 Department of Endocrinology-Diabetology-Nutrition, AP-HP, Avicenne Hospital, Bobigny, France; 5 Equipe de Recherche en Epidémiologie Nutritionnelle (EREN), Université Sorbonne Paris Nord and Université Paris Cité Inserm, INRAE, CNAM, Centre of Research in Epidemiology and StatisticS (CRESS), Bobigny, France; 6 Service de Diabétologie, Endocrinologie et Nutrition, Hôpital Paris Saint-Joseph, Paris, France; 7 Université de Reims Champagne-Ardenne, CHU Reims, CRESTIC, Service d’Endocrinologie Diabète Nutrition, Reims, France; 8 Aix Marseille Univ, APHM, Inserm, INRAE, C2VN, University Hospital La Conception, Department of Nutrition, Metabolic Diseases and Endocrinology, Marseille, France; 9 Endocrinologue-Diabétologue, Blanquefort, France; 10 Department of Endocrinology, Diabetology and Nutrition, Centre Hospitalier du Pays d’Aix, Aix en Provence, France; 11 Service de Endocrinologie - Diabétologie - Nutrition, Hôpital Saint-Joseph, Marseille, France; 12 Centre Universitaire de Diabétologie et de ses Complications, AP-HP, Hôpital Lariboisière, Paris, France; 13 Service de Diabétologie, Hôpital Européen, Marseille, France; 14 Department of Diabetology, Clinique Pasteur, Toulouse, France; 15 Institut Necker-Enfants Malades, Inserm U1151, CNRS UMR 8253, IMMEDIAB Laboratory, Paris, France

**Keywords:** Continuous glucose monitoring, Impaired awareness of hypoglycaemia, Severe hypoglycaemia, Time below range, Type 1 diabetes

## Abstract

**Aims/hypothesis:**

Severe hypoglycaemia events (SHE) remain frequent in people with type 1 diabetes despite advanced diabetes technologies. We examined whether time below range (TBR) 3.9 mmol/l (70 mg/dl; TBR70) or 3.0 mmol/l (54 mg/dl; TBR54) is associated with future SHE risk and whether impaired awareness of hypoglycaemia (IAH) modifies this relationship.

**Methods:**

We analysed data from participants in the Study of the French-speaking Society of Type 1 Diabetes (SFDT1) who used continuous glucose monitoring. IAH was assessed using the Gold Score (≤2, no IAH; 3, undetermined; ≥4, IAH). SHE frequency was self-reported 12 months after inclusion. We analysed associations between TBR and SHE using logistic regression models adjusted for age, sex, social vulnerability and insulin treatment, including TBR–IAH interactions. We performed spline analyses to explore non-linear patterns.

**Results:**

One-year incidence of SHE was 11.7% among 848 participants (mean ± SD age 41.6 ± 13.3 years; 53.8% female sex, HbA_1c_ 57.2 ± 10.9 mmol/mol [7.4 ± 1.0%]). Incidence by TBR70 was 12.1% for ≤1%, 10.2% for 1.1–3.9%, 10.6% for 4–6%, and 14.6% for >6%. Only those with TBR70 >6% and IAH had a significantly higher SHE risk (OR 3.32 [95% CI 1.40, 7.82]) compared with TBR70 ≤1% and no IAH. For TBR54, SHE incidence was 11.0% and 13.3% for categories <1% and ≥1%, respectively. Similarly, only individuals with TBR54≥1% and IAH had increased SHE risk (OR 2.99 [95% CI 1.46, 5.92]). Spline analysis showed low, stable SHE risk across TBR70 values in participants without IAH, with a non-linear pattern only in those with IAH.

**Conclusions/interpretation:**

TBR alone is not discriminative for high-risk SHE but combining TBR with hypoglycaemia awareness status identifies those at the highest risk for both TBR70 and TBR54.

**Trial registration:**

ClinicalTrials.gov NCT04657783

**Graphical Abstract:**

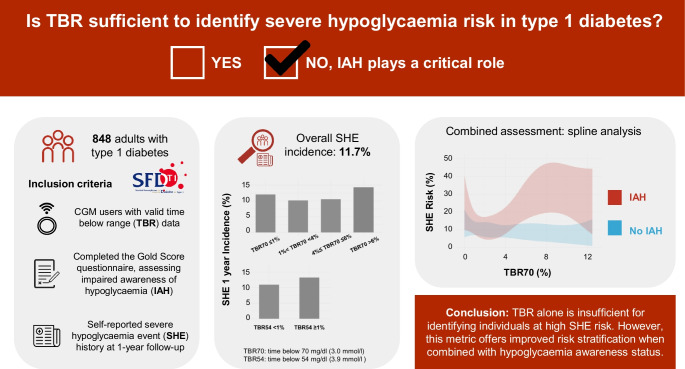

**Supplementary Information:**

The online version of this article (10.1007/s00125-025-06536-x) contains peer-reviewed but unedited supplementary material.


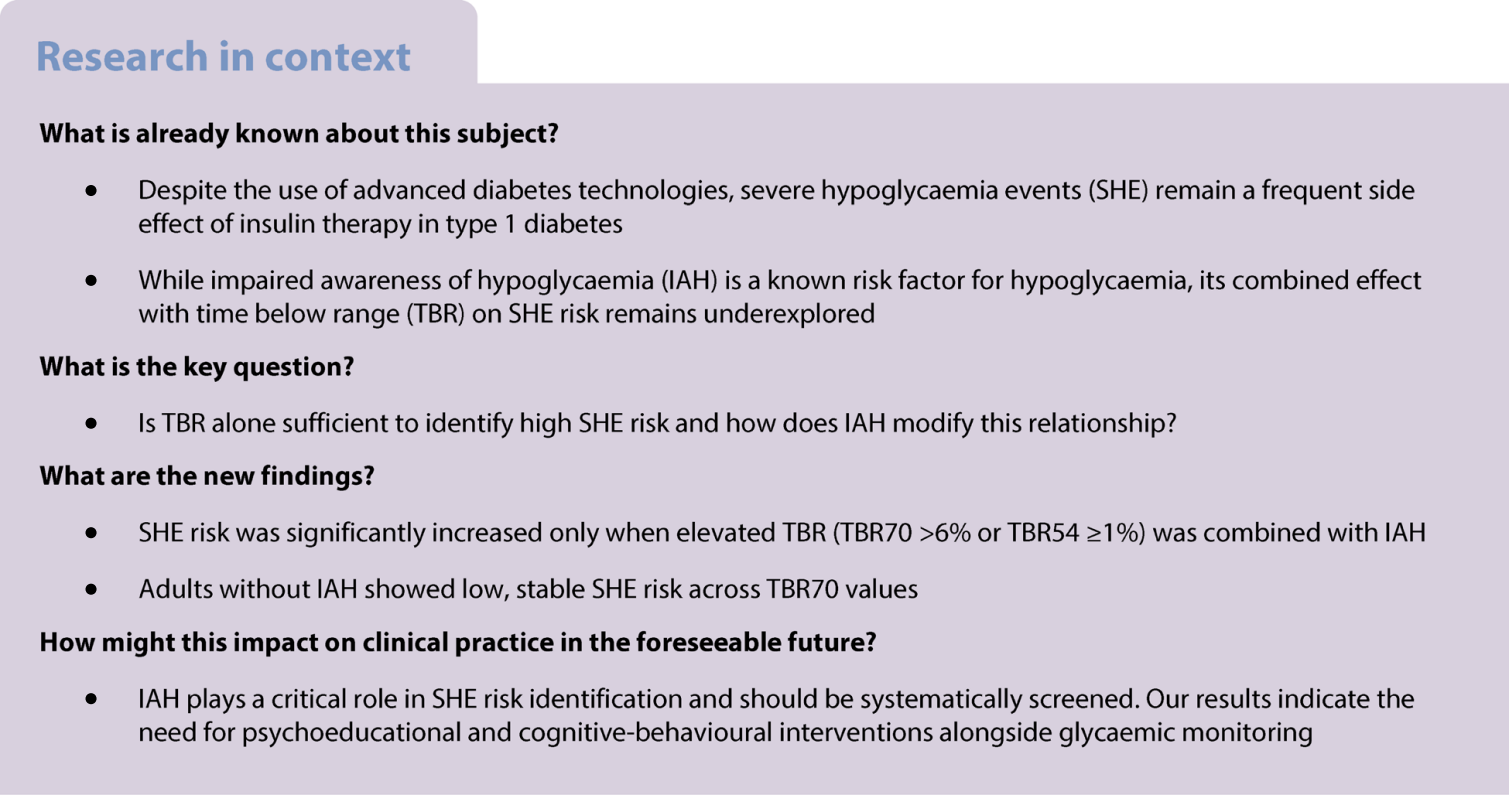


## Introduction

Severe hypoglycaemia events (SHE) are defined as hypoglycaemic events associated with severe cognitive impairment requiring assistance from another person for recovery [1]. Despite technological advances such as continuous glucose monitoring (CGM) and automated insulin delivery (AID), which are proven to improve glycaemic targets, the prevalence of SHE remains unacceptably high [2, 3]. Managing SHE is crucial for preventing acute health risks, including seizures, unconsciousness and cardiovascular events, as well as mitigating the long-term psychological burden and cognitive function declines associated with type 1 diabetes [4–6].

Key markers associated with SHE risk focus on CGM-derived metrics such as percentage of time below range (TBR). This metric captures periods where blood glucose falls below specific thresholds, including 3.9 mmol/l (70 mg/dl; TBR70) and 3.0 mmol/l (54 mg/dl; TBR54). Since 2019, clinical guidelines have recommended that people with type 1 diabetes maintain TBR70 below 4% and TBR54 below 1% [7].

Recent studies suggest that prolonged periods spent below these thresholds are associated with an increased likelihood of SHE [8–10]. However, evidence of TBR’s accuracy in identifying high-risk individuals remains inconclusive. A recent large-scale study involving over 5000 individuals with type 1 diabetes found that the standard international TBR70 <4% lacks sufficient discriminatory power to reliably predict SHE [11]. Another study on younger individuals with type 1 diabetes observed numerous TBR events, with only one self-reported SHE [12], suggesting that frequent mild and moderate hypoglycaemia, as indicated by TBR70 and TBR54, respectively, does not necessarily correlate with SHE occurrences.

Recurrent hypoglycaemia diminishes counterregulatory hormone responses and the ability to recognise hypoglycaemia symptoms, causing impaired awareness of hypoglycaemia (IAH) [2]. The prevalence of IAH follows the same pattern as SHE: despite the use of technology, the prevalence of IAH remains high [13]. Interestingly, a recent cross-sectional study involving more than 2000 participants with type 1 diabetes even showed higher IAH rates among CGM users (31%) compared with non-users (26%) [2]. The persistent prevalence of IAH poses a significant risk for SHE, with at least two cross-sectional studies in type 1 diabetes showing an association between them [3, 14]. Reflecting this risk, IAH is included in ADA clinical practice recommendations as part of the high-risk criteria for hypoglycaemia [15], underscoring its importance in identifying individuals at greater risk of SHE.

Recent evidence suggests that combining these metrics could improve the predictive accuracy of SHE [11]. However, to our knowledge, the extent to which IAH modifies the relationship between TBR and SHE risk remains underexplored, as it is unknown how these measures can work synergistically to improve the accuracy of SHE risk prediction and guide better clinical decision-making.

Therefore, this study aims to further investigate whether TBR alone is enough to identify SHE risk and to explore the joint effect of IAH in this relationship. A better understanding of this interaction could improve SHE risk stratification and support more-targeted interventions for individuals with type 1 diabetes at the highest risk.

## Methods

### Study design

This work used data from the Study of the French-speaking Society of Type 1 Diabetes (SFDT1) [16]. This ongoing cohort covers the entire French territory, including participants of diverse ages, sexes, ethnicities and socioeconomic backgrounds. All these variables were recorded except for ethnicity due to national ethics regulations in France. The SFDT1 study collects data on sex (female or male) but not on gender. It combines data based on monthly electronic patient-reported outcomes (ePROs, filled remotely), face-to-face interviews (F2F), physical examinations, clinical assessments, blood sample analysis and CGM measures (ClinicalTrials.gov registration no. NCT04657783). Sanoïa supported the study protocol design, ethical procedures and implementation and orchestrated the data collection and data flows on its secure platform. The study was approved by the ethics committee CPP Ouest V-RENNES (no. ID-RCB: 2019A01681-56) in December 2019.

**Fig. 1:**
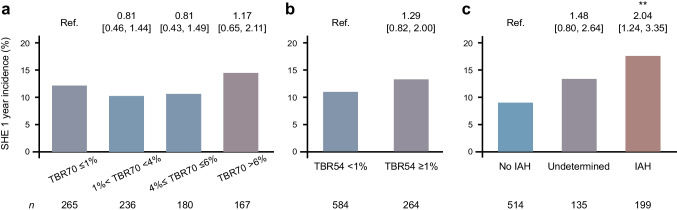
Relationship between SHE 1 year incidence and TBR70 categories (**a**), TBR54 categories (**b**) and IAH (**c**). Bars represent the percentage of SFDT1 participants with at least one SHE during the 1 year follow-up. *n* indicates the number of individuals in each TBR or IAH category. Associations were investigated using logistic regression models adjusted for age, sex, social vulnerability and insulin treatment. OR and 95% CI are displayed at the top of each bar. No TBR70 category or TBR54 event was associated with an increased risk of SHE whereas IAH alone was associated with an increased risk of SHE. **p<0.01

The current analysis included participants enrolled between December 2020 and January 2025. Besides the inclusion criteria of this cohort [16], the present study included SFDT1 participants with CGM data who filled in the Gold questionnaire (F2F, assessing IAH [17]) at baseline, as well as those who self-reported the occurrence or absence of SHE in the following 12 months (ePRO 12M, see electronic supplementary material [ESM] Fig. 1).

### Variables collected

Unless otherwise specified, individual characteristics, clinical variables, CGM data and information on diabetes-related complications were collected at baseline, during the inclusion visit.

Individual characteristics included age, sex (from medical records), higher education (defined as two or more years after high school) and social vulnerability measured using the EPICES (*Evaluation de la Précarité et des Inégalités de santé dans les Centres d’Examens de Santé*, F2F) score, with a cut-off of ≥30.17 indicating vulnerability [18]. Alcohol consumption was categorised as never, yes but no excess, or yes and excess (with excess being defined as more than three drinks for female participants and four for male participants on any single day) [19]. Smoking status was classified as never, past smoker or current smoker.

Clinical indicators included diabetes duration, insulin treatment (categorised as multiple daily injections [MDI], insulin pump, insulin pump combined with another device [open-loop], or AID system), BMI, last HbA_1c_, systolic BP and heart rate. Lipid profile measurements included total cholesterol, HDL-cholesterol, LDL-cholesterol and triglycerides. Insulin treatment was recorded both at baseline (inclusion visit) and at follow-up (ePRO 12M); however, because the timing of any treatment change relative to the occurrence of SHE during follow-up was unknown, these data were analysed descriptively only.

CGM-derived data included TBR70, TBR54, time in range (TIR, % time between 3.9 and 10.0 mmol/l [70 and 180 mg/dl]), time above range (TAR, above 10.0 mmol/l [180 mg/dl]) and CV (%). The glycaemic risk index (GRI) was also used to evaluate the risk of acute hypo- and hyperglycaemic events based on the following four CGM variables: TBR54; TBR70; TAR; and % time over 13.9 mmol/l (250 mg/dl) [20]. The CGM data collection corresponded to a 2 week period and was required to capture at least 70% of the data, and the sum of TBR, TAR and TIR had to be between 98% and 102% to ensure data consistency [7]. Additionally, the CGM device brand (Abbott, Dexcom or Medtronic) was recorded at baseline.

Diabetes-related complications were categorised into acute and long-term complications. Acute complications included SHE and diabetic ketoacidosis (DKA). Incident SHE was self-reported by participants 12 months post-inclusion by answering the question: ‘Did you have one or more severe hypoglycaemia events (requiring someone’s help to restore sugar levels)?’. Additionally, prior history of SHE was collected at baseline to analyse pre-existing risk. Long-term complications included CVD, retinopathy, nephropathy and neuropathy. CVD was defined as a history of stable angina, acute coronary syndrome, coronary artery bypass graft, stroke, transient ischaemic attack or hospitalisation for heart failure. Retinopathy (any degree) was diagnosed based on participant interviews, retinography, fundus examination and/or an ophthalmologist’s consultation report. Nephropathy was identified based on an albumin/creatinine ratio >3 mg/mmol or an eGFR <60 ml/min per 1.73 m^2^ [21]. Neuropathy was assessed through physical examination using the Michigan Neuropathy Screening Instrument, with a diagnosis threshold >2 [22].

Hypoglycaemia awareness status was assessed at baseline using the Gold Score, where a score of <3 reflects no IAH and >3 indicates IAH (a score of 3 was considered undetermined) [17]. Other patient-reported outcomes included fear of hypoglycaemia, measured at baseline (F2F) with the short form of the Hypoglycaemia Fear Survey (HFS-II), providing separate behavioural and affective scores ranging from 0 to 16 [23]. Diabetes-dependent quality of life, measured remotely 1 month after inclusion (ePRO 1M) using the Audit of Diabetes-Dependent Quality of Life (ADDQoL), provides an average weighted impact score on several aspects of life, ranging from −9 (worst) to +3 [24]. Treatment burden was evaluated using the Treatment Burden Questionnaire (TBQ) at month 2 (ePRO 2M), with a total score ranging from 0 to 150 [25]. Diabetes-related distress was assessed at month 3 (ePRO 3M) using the Problem Areas in Diabetes (PAID) questionnaire [26], where a score of PAID ≥40 indicates high distress [27].

All questionnaires were validated and adapted to French. Further details about the study setting and timeline are provided in ESM Fig. 1.

### Missing data and multiple imputation

Some data were missing for some of the variables (ESM Table 1). For all variables except those derived from CGM and for the variables of interest (TBR, IAH and SHE), assuming a missing-at-random mechanism, we applied multiple imputations using the chained-equation approach with the ‘mice’ R package (version 3.16.0) [28].

We generated 35 imputed datasets based on the maximum percentage of missing data, following White et al’s rule of thumb [29]. The number of iterations was 20 and the number of predictor variables was 25, in agreement with Van Buuren [30]. We used an imputation model with the best predictors of missing data, by including relevant confounders and excluding highly correlated variables. We evaluated convergence with means, variances and visual representations. Descriptive variables were pooled by calculating each individual’s mean and mode of all imputed values [31]. The estimates were pooled, and the CIs were calculated according to Rubin’s rules.

### Statistical analyses

All statistical analyses were performed using R software version 4.4.1 (https://cran.r-project.org/). To address clinical non-viability and potential errors, we removed outlier values for TBR70 and TBR54 using the IQR approach [32]. We set Q3 at the 85th percentile to adopt a less-conservative approach and ensure we kept meaningful values. We validated the outliers using domain knowledge from clinicians. We stratified TBR70 values based on mean and quantiles (1.0, 3.9, 6.0), ensuring alignment with the recognised 4% threshold. We stratified TBR54 based on the established 1% threshold.

We present the characteristics of the study population overall and stratified by incident SHE (at least one event during the 1 year of follow-up). We used means and SDs for continuous and normally distributed variables and medians (IQR) otherwise. Categorical variables were reported as counts (%).

Primary analyses were conducted using logistic regression models with SHE as the outcome and TBR or IAH as the main determinants. The models were adjusted for age, sex, insulin treatment and social vulnerability. Interaction terms between TBR and IAH were included to examine how IAH status modified the relationship between TBR and SHE risk across different TBR levels, aiming to identify high-risk profiles.

In addition, we applied logistic regression with splines (3 *df*) to capture potential non-linear relationships between TBR and SHE risk. This approach allowed us to explore how TBR values evolved in individuals with and without IAH, providing a more detailed assessment of risk variation.

We performed a sensitivity analysis to evaluate potential selection bias. This involved comparing the characteristics of included participants with those excluded, particularly non-completers of the follow-up questionnaire (ePRO 12M). We also examined whether a history of SHE at baseline was associated with increased risk of SHE at follow-up, adjusting for age, sex, insulin treatment and social vulnerability.

## Results

### Study population and baseline characteristics

We analysed data from 848 adults with type 1 diabetes in France (for flowchart, see ESM Fig. 2). The mean ± SD age was 41.6 ± 13.3 years, with a diabetes duration of 23.8 ± 13.7 years; 456 (53.8%) participants were female (Table 1). Among SFDT1 respondents, 591 (69.7%) used insulin pumps, of which 152 (25.7%) used an AID. The median (IQR) HbA_1c_ level was 56.3 mmol/mol (50.8–62.8) (7.3% [6.8–7.9]); mean ± SD 57.2 ± 10.9 mmol/mol (7.4 ± 1.0%). A total of 501 (59.1%) participants achieved the target TBR70 <4%, while 584 (68.9%) achieved TBR54 <1%. The prevalence of IAH was 23.5%.

**Table 1:** Clinical phenotyping of SFDT1 cohort according to SHE incidence (at least one event between baseline and 12 months of follow-up)

Baseline characteristic	All (*N*=848)	No SHE at 1 year (*n*=749)	SHE at 1 year (*n*=99)	*p* value
No. of SHE, *n* (%)				
1			41 (41.4)	
2 or 3			35 (35.4)	
>3			23 (23.2)	
Individual				
Age, years	41.60 ± 13.31	41.60 ± 13.34	41.66 ± 13.15	0.967
Female sex, *n* (%)	456 (53.8)	397 (53.0)	59 (59.6)	0.259
Diabetes duration, years	23.79 ± 13.74	23.57 ± 13.80	25.48 ± 13.21	0.192
Higher education, *n* (%)	609 (71.8)	542 (72.4)	67 (67.7)	0.392
Socially vulnerable, *n* (%)	138 (16.3)	112 (15.0)	26 (26.3)	0.007
Alcohol consumption, *n* (%)				0.190
Never	212 (25.0)	180 (24.0)	32 (32.3)	
Yes, but not in excess	595 (70.2)	533 (71.2)	62 (62.6)	
Yes, in excess	41 (4.8)	36 (4.8)	5 (5.1)	
Smoking status, *n* (%)				0.714
Never	488 (57.5)	434 (57.9)	54 (54.5)	
Past smoker	228 (26.9)	201 (26.8)	27 (27.3)	
Current smoker	132 (15.6)	114 (15.2)	18 (18.2)	
Clinical				
CGM brand, *n* (%)				0.316
Abbott	596 (70.3)	528 (70.5)	68 (68.7)	
Dexcom	108 (12.7)	91 (12.1)	17 (17.2)	
Medtronic	144 (17.0)	130 (17.4)	14 (14.1)	
Insulin treatment, *n* (%)				0.499
MDI	257 (30.3)	228 (30.4)	29 (29.3)	
Only pump	182 (21.5)	162 (21.6)	20 (20.2)	
Open-loop	257 (30.3)	221 (29.5)	36 (36.4)	
AID	152 (17.9)	138 (18.4)	14 (14.1)	
Insulin treatment change, *n* (%)^a^				0.865
No change	413 (48.7)	368 (49.1)	45 (45.5)	
AID initiation	153 (18.0)	135 (18.0)	18 (18.2)	
AID discontinuation	41 (4.8)	35 (4.7)	6 (6.1)	
Other change	241 (28.4)	211 (28.2)	30 (30.3)	
BMI	25.51 (23.05–28.73)	25.56 (23.11–29.00)	25.21 (22.96–28.39)	0.387
HbA_1c_, mmol/mol	56.27 (50.81–62.83)	56.27 (50.81–62.83)	56.27 (48.68–66.11)	0.796
HbA_1c_, %	7.30 (6.80–7.90)	7.30 (6.80–7.90)	7.30 (6.61–8.20)	
TBR54 <1%, *n* (%)	584 (68.9)	520 (69.4)	64 (64.6)	0.396
TBR70, %	3.00 (1.00–6.00)	3.00 (1.00–5.20)	3.00 (1.00–6.00)	0.948
TBR70 <4%, *n* (%)	501 (59.1)	445 (59.4)	56 (56.6)	0.665
TIR, %	59.99 ± 15.66	60.06 ± 15.44	59.48 ± 17.33	0.731
TAR, *n* (%)	36.12 (16.22)	36.05 (16.00)	36.64 (17.88)	0.731
GRI, %	57.30 ± 28.45	57.06 ± 27.67	59.16 ± 33.91	0.490
CV, %	37.60 (33.90– 41.00)	37.54 (33.90– 40.90)	38.20 (34.20– 41.50)	0.708
Systolic BP, mmHg	124.36 ± 16.93	124.55 ± 17.08	122.94 ± 15.80	0.373
Heart rate, beats/min	74.97 ± 13.55	74.50 ± 13.40	78.47 ± 14.24	0.006
Total cholesterol, mmol/l	4.50 (3.90–5.07)	4.49 (3.89–5.07)	4.61 (4.08–4.99)	0.301
LDL-cholesterol, mmol/l	2.55 ± 0.81	2.55 ± 0.82	2.56 ± 0.75	0.951
HDL-cholesterol, mmol/l	1.56 (1.32–1.81)	1.55 (1.32–1.81)	1.63 (1.41–1.86)	0.083
Triglycerides, mmol/l	0.78 (0.63–1.01)	0.78 (0.63–1.00)	0.77 (0.62– 1.05)	0.937
Complications				
Acute complications (at least one event during the previous year), *n* (%)				
Severe hypoglycaemia	68 (8.0)	43 (5.7)	25 (25.3)	<0.001
Diabetic ketoacidosis	27 (3.2)	23 (3.1)	4 (4.0)	0.832
Long-term complications, *n* (%)				
CVD	41 (4.8)	40 (5.3)	1 (1.0)	0.101
Retinopathy	264 (31.1)	231 (30.8)	33 (33.3)	0.698
Nephropathy	210 (24.8)	183 (24.4)	27 (27.3)	0.623
Neuropathy	305 (36.0)	262 (35.0)	43 (43.4)	0.125
Patient-reported outcomes				
ADDQoL average weighted impact score [−9 to 3]	−2.55 ± 1.66	−2.46 ± 1.60	−3.20 ± 1.93	<0.001
Gold Score	2.00 (1.00–3.00)	2.00 (1.00–3.00)	3.00 (2.00–4.50)	0.007
Hypoglycaemia awareness, *n* (%)				0.005
Aware	514 (60.6)	468 (62.5%)	46 (46.5%)	
Undetermined	135 (15.9)	117 (15.6%)	18 (18.2%)	
Impaired	199 (23.5)	164 (21.9%)	35 (35.4%)	
Affective fear of hypoglycaemia score [0–16]	4.81 ± 3.34	4.67 ± 3.27	5.92 ± 3.69	<0.001
Behavioural avoidance of hypoglycaemia score [0–16]	6.73 ± 2.89	6.69 ± 2.87	7.05 ± 3.06	0.239
Treatment burden score [0–150]	60.21 ± 28.42	58.89 ± 27.56	70.19 ± 32.75	<0.001
Diabetes distress PAID score, total [0–100]	39.65 ± 20.41	38.73 ± 20.03	46.58 ± 22.03	<0.001

Data are shown as mean ± SD, median (IQR) or *n* (%); values within square brackets indicate minimum–maximum values for that variable

^a^The timing of any treatment change relative to the occurrence of SHE during follow-up was unknown

*p* values were calculated using *t* test or Wilcoxon test to compare group differences with continuous outcomes and χ^2^ test to compare categorical variables

ADDQoL, Audit of Diabetes-Dependent Quality of Life

### Characteristics of participants lost to follow-up

Participants who did not complete the follow-up questionnaire at month 12 (*n*=1079) were predominantly male, younger, had higher levels of hyperglycaemia, and most used MDI as their insulin treatment (ESM Table 2).

### Comparison of participants with and without incident SHE

Table 1 presents a comparative analysis of baseline characteristics between participants who did not report any SHE during the follow-up year (*n*=749, 88.3%) and those who experienced at least one episode (*n*=99, 11.7%). At baseline, individuals in the SHE group were more socially vulnerable, had elevated heart rate and higher psychological burden, characterised by poorer diabetes-related quality of life, more significant treatment burden and higher diabetes-related distress. Interestingly, only affective fear of hypoglycaemia, but not behavioural avoidance of hypoglycaemia, was significantly increased in the SHE group. No significant differences in sex or age were observed. Notably, the prevalence of SHE in the year before baseline was significantly higher in this group (25.3% vs 5.7% in the no-SHE group). Consistently, baseline SHE history was a strong predictor of incident SHE (OR 6.11 [95% CI 3.43, 10.79], *p*<0.001). There was no significant difference in the distribution of CGM devices or insulin treatment modalities between the two groups.

ESM Table 3 and ESM Table 4 present a comparative analysis of baseline characteristics across TBR70 and TBR54 categories, respectively.

### TBR and IAH as SHE risk predictors

#### Individual assessment

Figure 1 illustrates the prevalence of participants with incident SHE across TBR70 (Fig. 1a), TBR54 (Fig. 1b) and IAH categories (Fig. 1c), presenting the number of individuals (*n*) in each category along OR and 95% CI from logistic regression models.

The prevalence of SHE was similar across TBR70 categories: 12.1% for TBR70 ≤1%, 10.2% for TBR70 1.1–3.9%, 10.6% for TBR70 4–6%, and 14.6% for TBR70 >6%. Similarly, for TBR54, the prevalence of SHE was 11.0% and 13.3% for TBR54 <1% and ≥1%, respectively. In contrast, SHE prevalence varied across IAH categories: 8.9% in individuals without IAH; 13.3% in those with undetermined status; and 17.6% in the IAH group. Notably, individuals with IAH had a statistically significant association with SHE compared with those without IAH (OR 2.04 [95% CI 1.24, 3.35], *p*<0.01).

#### Combined assessment

**Fig. 2:**
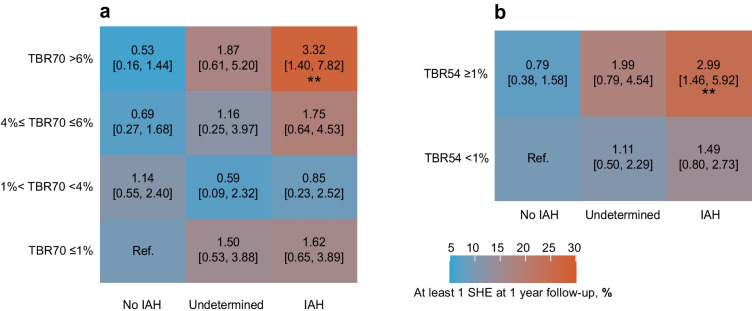
Heatmap showing the percentage of SFDT1 participants with at least one SHE during the 1 year follow-up across IAH TBR70 (**a**) or TBR54 (**b**) interaction groups. Associations were investigated using logistic regression models adjusted for age, sex, social vulnerability and insulin treatment. OR and 95% CI are displayed. Only the TBR70 >6%–IAH and TBR54 ≥1%–IAH interactions were associated with an increased risk of SHE. **p<0.01

Logistic regression models demonstrated that TBR70 alone was not a strong predictor of SHE across categories. However, when incorporating the interaction with IAH, individuals with both TBR70 >6% and IAH had a significantly increased SHE risk compared with those with TBR70 ≤1% and no IAH (OR 3.32 [95% CI 1.40, 7.82], *p*<0.01) (Fig. 2). Similarly, participants with TBR54 ≥1% and IAH had a significantly higher SHE risk compared with those with TBR54 <1% and no IAH (OR 2.99 [95% CI 1.46, 5.92], *p*<0.01).

**Fig. 3:**
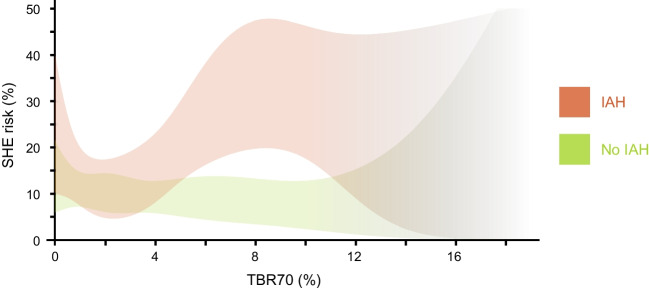
Spline logistic regression analysis of the predicted probability (risk) of an SHE during the follow-up year based on TBR70 at baseline in individuals with no IAH vs with IAH. The plot displays the 95% CI of the predicted values

Figure 3 shows the curve derived from spline logistic regression models, highlighting distinct risk patterns between individuals with and without IAH. The plot displays the 95% CIs of the predicted values. ESM Fig. 3 presents the same analysis but based on TBR54.

Among individuals without IAH, the predicted SHE risk remained relatively low (~10%) and stable across the TBR70 range (Fig. 3). In contrast, for those with IAH, the probability of SHE showed a non-linear pattern, with a notable increase as TBR70 exceeded ~6%. Particularly within the 8–9% TBR70 range, the 95% CI for SHE risk ranged from a minimum of 20% to nearly 50%. The widening CIs at higher TBR70 levels reflect increased uncertainty due to fewer observations in this range.

For TBR54 (ESM Fig. 3), due to limited data, the uncertainty was even greater, preventing the identification of specific trends. However, around TBR54 =1%, a distinction is visible between those with IAH and those without IAH, with the 95% CI for SHE risk ranging from approximately 5–15% to 15–35%, respectively.

## Discussion

### Main findings

This study highlights that TBR70 and TBR54 alone are insufficient for identifying individuals at high SHE risk. However, these metrics offer improved risk stratification when combined with hypoglycaemia awareness status. Specifically, TBR70 >6% and TBR54 ≥1% were associated with significantly higher SHE risk in individuals with IAH.

### SHE prevalence

The UK-based study recently conducted by Deshmukh et al [11] stands out due to its large sample size of 5029 participants from the Association of British Clinical Diabetologists audit (97% with type 1 diabetes). Our study had a higher prevalence of SHE (12% vs 4%) and IAH (24% vs 17%). Notably, participants in our cohort had better hypoglycaemic management at baseline (59% vs 42% meeting the TBR70 <4% target) but SHE prevalence remained higher. Differences in population characteristics and study design may explain this discrepancy. Our study assessed IAH prevalence at baseline whereas Deshmukh et al measured it at follow-up. Given that IAH is a strong independent risk factor for SHE, the higher IAH prevalence in our cohort may have contributed to the increased SHE rate.

Further supporting our findings, studies using the T1D Exchange cohort in the USA reported higher SHE rates, with a prevalence of 20% [2], despite a high adoption of CGM and AID systems. This suggests that even with advanced diabetes technologies, a substantial proportion of individuals with type 1 diabetes continue to experience SHE, reinforcing the importance of factors beyond glucose metrics. While use of an AID system is indeed associated with improved glycaemic outcomes, as evidenced by a sharp decline in its use among those with TBR70 >6% (*p*<0.001, ESM Table 3), it is not sufficient on its own, underscoring the critical role of IAH. Consistent with our results, another T1D Exchange study also found IAH to be independently associated with higher SHE risk [3]. Moreover, a recent systematic review [33] reinforces that although AID systems may offer some advantages, psychoeducational interventions remain the cornerstone of IAH management.

Importantly, differences in SHE and IAH prevalence across studies may also come from variations in definitions and assessment methods (medical records vs self-assessment), as already noted in a 2017 review [34]. A T1D Exchange study showed that differences in SHE wording can impact self-reported prevalence [35]. Other studies also showed that IAH prevalence varies based on the assessment method [3, 36]. These discrepancies highlight the need for standardised criteria for both SHE and IAH assessments to improve cross-study comparability, risk stratification and clinical decision-making.

### SHE risk prediction

The stratification of TBR70 categories allowed us to go beyond the 4% threshold, revealing that individuals with TBR70 >6% and IAH have more than three times the odds of experiencing an SHE in the following year than those without IAH and TBR70 ≤1% (Fig. 2a). The same pattern was noted for those with TBR54 ≥1% and IAH. The ORs for both exceeded the point estimate of the risk associated with IAH alone (OR 2.04, *p*<0.01, Fig. 1c). However, these differences were not statistically significant. Nevertheless, the observed pattern suggests a potential combined effect beyond IAH alone, reinforcing the importance of considering both TBR and IAH together to improve SHE risk prediction.

Our spline logistic regression findings align with our logistic regression analysis, further confirming that in individuals with TBR70% >6%, there is a clear separation from those with vs without IAH (Fig. 3). We observed a significant increase in SHE risk between 6% and 9% TBR70, beyond which data become sparse, highlighting the need for future research to explore extreme TBR values. Interestingly, at TBR70 0%, individuals with IAH exhibited a wide 95% CI ranging from 10% to 40%, suggesting that some individuals who maintain ‘perfect’ hypoglycaemic management at baseline but also exhibit IAH may struggle to respond effectively to hypoglycaemic events. In contrast, the lowest SHE risk for individuals with IAH was around TBR70 2%, where the predicted probabilities overlapped with those predicted for no IAH. This suggests that a balanced glycaemic management, even in those with IAH, may help mitigate SHE risk.

Physiological research has validated the use of IAH assessments combined with CGM-derived thresholds to identify individuals with absent autonomic symptom responses during hypoglycaemic clamp testing (a surrogate for clinically significant IAH) [37]. These findings strengthen the clinical relevance of combining (self-reported) IAH and TBR metrics for SHE risk stratification.

### Beyond TBR and IAH

The psychological burden associated with future SHE is also an important topic. Participants who experienced SHE reported higher fear of hypoglycaemia and greater diabetes-related distress at baseline (*p*<0.001 for both, Table 1). This aligns with a European survey of 458 individuals with type 1 diabetes, in which 71% identified hypoglycaemia as their primary diabetes-related concern [38]. A Portuguese study also found that individuals with both IAH and SHE were more prone to behavioural dysfunction, indicating greater perceived limitations aspects of daily life due to diabetes [39]. Consistently, our findings show a significantly more negative perceived impact of diabetes on quality of life among individuals with SHE, as reflected by lower ADDQoL scores (*p*<0.001, Table 1). These findings underscore the need for psychoeducational interventions alongside glycaemic management strategies.

Notably, while fear of hypoglycaemia was significantly higher in those who later experienced SHE, behavioural avoidance was not. This suggests that emotional distress alone does not necessarily translate into preventive action. This disconnect may be linked to IAH, where individuals fear hypoglycaemia but fail to recognise and respond to it. Cognitive distortions may further contribute, as Cook et al [40] found that those with IAH were more likely to prioritise hyperglycaemia concerns than those with intact awareness. Similarly, those with recurrent SHE showed a tendency toward hyperglycaemia prioritisation, driven by the presence of both IAH and SHE [40]. Our findings support this pattern, with prior history of SHE at baseline being a strong predictor of incident SHE at follow-up.

Along with psychological factors, social vulnerability should also be considered. In line with findings from a study on individuals with type 1 diabetes [41], SHE was more prevalent in socially vulnerable participants in our cohort (*p*<0.01, Table 1). Importantly, a recent cross-sectional study [42] highlighted that both IAH and recent SHE were independently associated with socioeconomic deprivation, as well as with mental health symptoms including depression and anxiety. These findings highlight the role of socioeconomic and mental health factors in SHE risk, further supporting the importance of integrating psychosocial determinants of health into diabetes management strategies.

### Strengths and limitations

To evaluate the potential risk of selection bias, we conducted a descriptive analysis (ESM Table 2) that examined the characteristics of individuals who did not complete the follow-up questionnaire at month 12. The findings revealed a mix of factors associated with both higher SHE risk (e.g. greater social vulnerability) and lower SHE risk (e.g. lower TBR, a higher proportion of individuals without IAH). While no formal statistical adjustment was applied, these opposing effects suggest that the direction of potential bias in SHE prevalence estimates is uncertain. Further research is needed to fully assess the potential influence of non-responders. Additionally, our study includes data from a diverse range of centres across various regions of France and different healthcare settings, helping to mitigate the risk of selection bias. However, using self-reported questionnaires introduces the possibility of recall bias, which may affect the accuracy of SHE reporting. Another limitation is that although TBR was assessed at baseline and SHE was reported at 1 year follow-up, we could not explore how changes in TBR over time related to the timing of SHE occurrence. Finally, we analysed only sex (female or male) and not gender data. This limitation may affect the generalisability of our findings to individuals whose gender identity does not align with their biological sex.

Despite these limitations, a key strength of our study is its large and diverse sample, offering valuable insights into the interplay between TBR, IAH and SHE risk. Additionally, the temporal design of our study allows for a more robust assessment of SHE risk at 1 year follow-up. To our knowledge, this is the first analysis in type 1 diabetes to demonstrate how TBR and IAH interact synergistically to refine SHE risk stratification. Furthermore, using spline regression enabled us to move beyond conventional thresholds, uncovering non-linear risk patterns that provide a more nuanced understanding of SHE risk across different TBR levels.

### Future directions

In line with ADA Standards of Care [15], annual screening for IAH should be standard practice. The guidelines recommend that SHE warrant behavioural education, treatment adjustments and cognitive assessment. Given its strong link to SHE, we argue that IAH should also trigger these interventions to reduce hypoglycaemia risk. As previously suggested [33], a holistic clinical approach that integrates technology with educational, psychosocial and cognitive-behavioural strategies is essential to effectively address IAH and SHE risk.

We believe that future work should better study the longitudinal relationship between TBR, IAH and SHE. Studies should focus on capturing detailed temporal patterns of TBR and IAH, ideally with more granular time resolution, to better understand how fluctuations in glycaemic management relate to the risk of SHE and associated underlying mechanisms. It is also relevant to explore how the implementation of CGM devices with low-glucose alarms may help prevent SHE, particularly in individuals with IAH [43]. Such approaches should also consider behavioural responses to hypoglycaemia and individual treatment strategies to improve predictive models and guide more personalised interventions.

Importantly, research and care should move beyond the current 4% TBR70 threshold, as its validity requires further evaluation. A recent study has shown that less than half of the episodes of CGM detected hypoglycaemia were detected by the participants with type 1 diabetes, even in those with intact awareness of hypoglycaemia [44]. This finding raises the question of whether the 70 mg/dl (3.9 mmol/l) threshold itself should be re-evaluated, as most individuals may not experience hypoglycaemia symptoms at that level. It also highlights the implications of how IAH is defined.

While AID systems represent a major advancement in diabetes care, they do not yet fully prevent the occurrence of SHE. Future algorithms integrating deep learning approaches for ECG-based hypoglycaemia detection [45], or genetic data to improve errors in glucose prediction [46], may offer further improvements in SHE prevention and management.

An important area for future research is the standardisation of SHE and IAH definitions. As highlighted recently by Lin et al [35], a consensus is needed to establish clear, standardised definitions of both SHE and IAH. Establishing these standardised definitions would enhance the validity and generalisability of future research findings.

Finally, by improving risk identification and intervention strategies, our results provide valuable insights for clinicians and researchers working toward improved SHE prevention.

## Supplementary Information

Below is the link to the electronic supplementary material.

ESM (PDF 526 KB)

## Data Availability

Data used for this analysis is available for academic researchers under request submitted to the scientific committee of SFDT1 (cohorte.sfdt1@gmail.com). R scripts created for this analysis are available under request to the corresponding author.

## Abbreviations

ADDQoL: Audit of Diabetes-Dependent Quality of Life
AID: Automated insulin delivery
CGM: Continuous glucose monitoring
ePRO: Electronic patient-reported outcome
F2F: Face-to-face interview
GRI: Glycaemic risk index
IAH: Impaired awareness of hypoglycaemia
MDI: Multiple daily injections
PAID: Problem areas in diabetes
SFDT1: Study of the French-speaking Society of Type 1 Diabetes
SHE: Severe hypoglycaemia event(s)
TAR: Time above range
TBQ: Treatment Burden Questionnaire
TBR: Time below range
TBR54: TBR, below 3.0 mmol/l (54 mg/dl)
TBR70: TBR, below 3.9 mmol/l (70 mg/dl)
TIR: Time in range

## Authors’ relationships and activities

PC has received personal fees from Abbott, Dexcom, Insulet, Medtronic, Ypsomed, Sanofi, Lilly, Roche, Vertex and CML. EC has received grants or contracts from Abbott, AstraZeneca, Novartis, Novo Nordisk, Roche Diagnostics and Sanofi. EC also received consulting fees from Abbott, AlphaDiab, Bayer, AstraZeneca, DexCom, Lilly, LVL Medical, Medtronic, Novartis, Novo Nordisk, Roche Diabetes and Sanofi and received honoraria for lectures, presentations, speakers bureaus, manuscript writing or educational events from Abbott, Amgen, Bayer, AstraZeneca, Boehringer Ingelheim, Lilly, LVL medical, Medtronic, Novo Nordisk, Roche Diagnostics, Sanofi, Urgo, ViiV Healthcare (GSK) and Roche Diagnostics. EC received support for attending meetings and/or travel from AlphaDiab, Asdia, Lilly, Sanofi and Vitalaire. GF has provided advisory/speaking services for and/or has received research grants and/or speaker honoraria from Sanofi, MSD, MSDAvenir, Eli Lilly, Roche Diabetes Care, AstraZeneca, Danone Research, Diabeloop, Bristol Myers Squibb, L’Oréal R&D, AbbVie Pharmaceutical, Pfizer, Vitalaire and Akuity Care. J-PR received payment or honoraria for lectures, presentations, speakers bureaus, manuscript writing or educational events from Sanofi Aventis, MSD, Eli Lilly, Novo Nordisk, AstraZeneca, Abbott, Dexcom, AlphaDiab, Medtronic, Air Liquide Santé International and Sanofi. The authors declare that there are no other relationships or activities that might bias, or be perceived to bias, their work.

## References

[1] Ratner RE (2018) Hypoglycemia: new definitions and regulatory implications. Diabetes Technol Ther 20(S2):S250–S253. 10.1089/dia.2018.0113 29873525 10.1089/dia.2018.0113

[2] Sherr JL, Laffel LM, Liu J et al (2024) Severe hypoglycemia and impaired awareness of hypoglycemia persist in people with type 1 diabetes despite use of diabetes technology: results from a cross-sectional survey. Diabetes Care 47(6):941–947. 10.2337/dc23-1765 38295397 10.2337/dc23-1765 PMC11116910

[3] Lin YK, Ye W, Hepworth E et al (2025) Characterising impaired awareness of hypoglycaemia and associated risks through HypoA-Q: findings from a T1D Exchange cohort. Diabetologia 68:433–443. 10.1007/s00125-024-06310-5 39477881 10.1007/s00125-024-06310-5 PMC11837905

[4] Amiel SA (2021) The consequences of hypoglycaemia. Diabetologia 64(5):963–970. https://doi-org.proxy.bnl.lu/10.1007/s00125-020-05366-3 10.1007/s00125-020-05366-3 PMC8012317 33550443

[5] Rossi MC, Nicolucci A, Ozzello A et al (2019) Impact of severe and symptomatic hypoglycemia on quality of life and fear of hypoglycemia in type 1 and type 2 diabetes. Results of the Hypos-1 observational study. Nutr Metab Cardiovasc Dis 29(7):736–743. 10.1016/j.numecd.2019.04.009 31153746 10.1016/j.numecd.2019.04.009

[6] Jacobson AM, Ryan CM, Braffett BH et al (2021) Cognitive performance declines in older adults with type 1 diabetes: results from 32 years of follow-up in the DCCT and EDIC Study. Lancet Diabetes Endocrinol 9(7):436–445. 10.1016/S2213-8587(21)00086-3 34051936 10.1016/S2213-8587(21)00086-3 PMC8583716

[7] Battelino T, Danne T, Bergenstal RM et al (2019) Clinical targets for continuous glucose monitoring data interpretation: recommendations from the international consensus on time in range. Diabetes Care 42(8):1593–1603. 10.2337/dci19-0028 31177185 10.2337/dci19-0028 PMC6973648

[8] Harvengt A, Beckers M, Boutsen L, Costenoble E, Brunelle C, Lysy P (2023) Deep analysis of clinical parameters and temporal evolution of glycemic parameters based on CGM data for the characterization of severe hypoglycemia in a cohort of children and adolescents with type 1 diabetes. Nutrients 15(13):2957. 10.3390/nu15132957 37447282 10.3390/nu15132957 PMC10346427

[9] Karges B, Tittel SR, Bey A et al (2023) Continuous glucose monitoring versus blood glucose monitoring for risk of severe hypoglycaemia and diabetic ketoacidosis in children, adolescents, and young adults with type 1 diabetes: a population-based study. Lancet Diabetes Endocrinol 11(5):314–323. 10.1016/s2213-8587(23)00061-x 37004710 10.1016/S2213-8587(23)00061-X

[10] Hermanns N, Ehrmann D, Heinemann L, Freckmann G, Waldenmaier D, Calhoun P (2022) Real-time continuous glucose monitoring can predict severe hypoglycemia in people with type 1 diabetes: combined analysis of the HypoDE and DIAMOND trials. Diabetes Technol Ther 24(9):603–610. 10.1089/dia.2022.0130 35604794 10.1089/dia.2022.0130

[11] Deshmukh H, Wilmot EG, Choudhary P et al (2025) Time below range and its influence on hypoglycemia awareness and severe hypoglycemia: insights from the association of British clinical diabetologists study. Diabetes Care 48(3):437–443. 10.2337/dc24-1833 39746160 10.2337/dc24-1833 PMC11870288

[12] Hill H, Klaar P, Espes D (2023) Real-life data of hypoglycemic events in children and adolescents with type 1 diabetes. BMJ Open Diabetes Res Care 11(5):e003485. 10.1136/bmjdrc-2023-003485 37739421 10.1136/bmjdrc-2023-003485 PMC10533671

[13] Yu X, Fan M, Zhao X et al (2023) Prevalence of impaired awareness of hypoglycaemia in people with diabetes mellitus: A systematic review and meta-analysis from 21 countries and regions. Diabet Med 40(9):e15129. 10.1111/dme.15129 37143390 10.1111/dme.15129

[14] Lin YK, Hung M, Sharma A et al (2019) Impaired awareness of hypoglycemia continues to be a risk factor for severe hypoglycemia despite the use of continuous glucose monitoring system in type 1 diabetes. Endocr Pract 25(6):517. 10.4158/ep-2018-0527 30865520 10.4158/EP-2018-0527 PMC6771275

[15] American Diabetes Association Professional Practice Committee (2025) 6. Glycemic Goals and Hypoglycemia: Standards of Care in Diabetes-2025. Diabetes Care 48(1 Suppl 1):S128–S145. 10.2337/dc25-s006 39651981 10.2337/dc25-S006 PMC11635034

[16] Riveline JP, Vergés B, Detournay B et al (2022) Design of a prospective, longitudinal cohort of people living with type 1 diabetes exploring factors associated with the residual cardiovascular risk and other diabetes-related complications: The SFDT1 study. Diabetes Metab 48(3):101306. 10.1016/j.diabet.2021.101306 34813929 10.1016/j.diabet.2021.101306

[17] Gold AE, MacLeod KM, Frier BM (1994) Frequency of severe hypoglycemia in patients with type I diabetes with impaired awareness of hypoglycemia. Diabetes Care 17(7):697–703. 10.2337/diacare.17.7.697 7924780 10.2337/diacare.17.7.697

[18] Guilloteau A, Binquet C, Bourredjem A et al (2020) Social deprivation among socio-economic contrasted french areas: Using item response theory analysis to assess differential item functioning of the EPICES questionnaire in stroke patients. PLoS One 15(4):e0230661. 10.1371/journal.pone.0230661 32240217 10.1371/journal.pone.0230661 PMC7117693

[19] Alcohol Research: Current Reviews Editorial Staff (2018) Drinking patterns and their definitions. Alcohol Res 39(1):17–18 30557143 10.35946/arcr.v39.1.03 PMC6104961

[20] Klonoff DC, Wang J, Rodbard D et al (2022) A Glycemia Risk Index (GRI) of hypoglycemia and hyperglycemia for continuous glucose monitoring validated by clinician ratings. J Diabetes Sci Technol 17(5):1226–1242. 10.1177/19322968221085273 35348391 10.1177/19322968221085273 PMC10563532

[21] de Boer IH, Khunti K, Sadusky T et al (2022) Diabetes management in chronic kidney disease: a consensus report by the American Diabetes Association (ADA) and Kidney Disease: Improving Global Outcomes (KDIGO). Diabetes Care 45(12):3075–3090. 10.2337/dci22-0027 36189689 10.2337/dci22-0027 PMC9870667

[22] Feldman EL, Stevens MJ, Thomas PK, Brown MB, Canal N, Greene DA (1994) A practical two-step quantitative clinical and electrophysiological assessment for the diagnosis and staging of diabetic neuropathy. Diabetes Care 17(11):1281–1289. 10.2337/diacare.17.11.1281 7821168 10.2337/diacare.17.11.1281

[23] Gonder-Frederick LA, Schmidt KM, Vajda KA et al (2011) Psychometric properties of the hypoglycemia fear survey-ii for adults with type 1 diabetes. Diabetes Care 34(4):801–806. 10.2337/dc10-1343 21346182 10.2337/dc10-1343 PMC3064031

[24] Krzemińska S, Bąk E, Šáteková L, Polanská A, Hašová K, Laurinc M (2020) Comparison of Diabetes-Dependent Quality of Life (ADDQoL) in patients with T2DM in poland, The Czech Republic, and Slovakia. Diabetes Metab Syndr Obes 13:3773–3786. 10.2147/dmso.s273339 33116726 10.2147/DMSO.S273339 PMC7585271

[25] Tran V-T, Montori VM, Eton DT, Baruch D, Falissard B, Ravaud P (2012) Development and description of measurement properties of an instrument to assess treatment burden among patients with multiple chronic conditions. BMC Med 10:68. 10.1186/1741-7015-10-68 22762722 10.1186/1741-7015-10-68 PMC3402984

[26] Polonsky WH, Anderson BJ, Lohrer PA et al (1995) Assessment of diabetes-related distress. Diabetes Care 18(6):754–760. 10.2337/diacare.18.6.754 7555499 10.2337/diacare.18.6.754

[27] Dennick K, Sturt J, Speight J (2017) What is diabetes distress and how can we measure it? A narrative review and conceptual model. J Diabetes Complications 31(5):898–911. 10.1016/j.jdiacomp.2016.12.018 28274681 10.1016/j.jdiacomp.2016.12.018

[28] van Buuren S, Groothuis-Oudshoorn K (2011) mice: multivariate imputation by chained equations in R. J Stat Soft 45:1–67. 10.18637/jss.v045.i03

[29] White IR, Royston P, Wood AM (2011) Multiple imputation using chained equations: issues and guidance for practice. Stat Med 30(4):377–399. 10.1002/sim.4067 21225900 10.1002/sim.4067

[30] van Buuren S (2018) Flexible imputation of missing data, 2nd edn. CRC Press, New York

[31] Burns RA, Butterworth P, Kiely KM et al (2011) Multiple imputation was an efficient method for harmonizing the Mini-Mental State Examination with missing item-level data. J Clin Epidemiol 64(7):787–793. 10.1016/j.jclinepi.2010.10.011 21292440 10.1016/j.jclinepi.2010.10.011

[32] Dallah D, Sulieman H (2024) Outlier detection using the range distribution. In: Kamalov F, Sivaraj R, Leung HH (eds) Advances in mathematical modeling and scientific computing. ICRDM 2022. Trends in Mathematics. Birkhäuser, Cham. 10.1007/978-3-031-41420-6_57

[33] Yeoh E, Choudhary P, Nwokolo M, Ayis S, Amiel SA (2015) Interventions that restore awareness of hypoglycemia in adults with type 1 diabetes: a systematic review and meta-analysis. Diabetes Care 38(8):1592–1609. 10.2337/dc15-0102 26207053 10.2337/dc15-0102

[34] Pedersen-Bjergaard U, Thorsteinsson B (2017) Reporting severe hypoglycemia in type 1 diabetes: facts and pitfalls. Curr Diab Rep 17(12):131. 10.1007/s11892-017-0965-1 29080929 10.1007/s11892-017-0965-1

[35] Lin YK, Ye W, Hepworth E, Ang L, Amiel SA, Fisher SJ (2025) Evaluating the impact of severe hypoglycaemia definition wording on severe hypoglycaemia history assessment. Diabet Med 42(4):e15513. 10.1111/dme.15513 39797557 10.1111/dme.15513 PMC11929560

[36] Michalak M, Zozulińska-Ziółkiewicz D, Michalak M, Cieluch A, Michalski M, Araszkiewicz A (2025) Efficacy of selected scales in the assessment of impaired awareness of hypoglycemia in adults with type 1 diabetes. Pol Arch Intern Med 135(3):16945. 10.20452/pamw.16945 39912801 10.20452/pamw.16945

[37] Flatt AJ, Chen E, Peleckis AJ et al (2022) Evaluation of clinical metrics for identifying defective physiologic responses to hypoglycemia in long-standing type 1 diabetes. Diabetes Technol Ther 24(10):737–748. 10.1089/dia.2022.0103 35758724 10.1089/dia.2022.0103 PMC9529296

[38] Penfornis A, Down S, Seignez A, Vives A, Bonnemaire M, Kulzer B (2025) European survey on adults with type 1 diabetes and their caregivers: insights into personal experience and needs for improving diabetes care. Diabetes Ther 16(3):471–484. 10.1007/s13300-024-01685-5 39883287 10.1007/s13300-024-01685-5 PMC11867988

[39] Sepúlveda E, Poínhos R, Nata G et al (2025) Relationship between severe hypoglycemia or impaired awareness of hypoglycemia and diabetes-related health status, global cognition and executive functions in adults with type 1 diabetes without severe anxiety or depression. Diabetes Res Clin Pract 221:112004. 10.1016/j.diabres.2025.112004 39805380 10.1016/j.diabres.2025.112004

[40] Cook AJ, DuBose SN, Foster N et al (2019) Cognitions associated with hypoglycemia awareness status and severe hypoglycemia experience in adults with type 1 diabetes. Diabetes Care 42(10):1854–1864. 10.2337/dc19-0002 31391200 10.2337/dc19-0002

[41] Baxter F, Baillie N, Dover A, Stimson RH, Gibb F, Forbes S (2024) A cross-sectional questionnaire study: Impaired awareness of hypoglycaemia remains prevalent in adults with type 1 diabetes and is associated with the risk of severe hypoglycaemia. PLOS ONE 19(6):e0297601. 10.1371/journal.pone.0297601 38875308 10.1371/journal.pone.0297601 PMC11178233

[42] Zammitt NN, Forbes S, Inkster B et al (2025) Predictors of impaired awareness of hypoglycaemia and severe hypoglycaemia in adults with type 1 diabetes. Diabet Med 19:e70074. 10.1111/dme.70074 10.1111/dme.70074 PMC12434435 40386839

[43] Oriot P, Hermans MP (2023) Intermittent-scanned continuous glucose monitoring with low glucose alarms decreases hypoglycemia incidence in middle-aged adults with type 1 diabetes in real-life setting. J Diabetes Complications 37(2):108385. 10.1016/j.jdiacomp.2022.108385 36603333 10.1016/j.jdiacomp.2022.108385

[44] Divilly P, Martine-Edith G, Zaremba N et al (2024) Relationship between sensor-detected hypoglycemia and patient-reported hypoglycemia in people with type 1 and insulin-treated type 2 diabetes: the hypo-METRICS study. Diabetes Care 47(10):1769–1777. 10.2337/dc23-2332 39207738 10.2337/dc23-2332 PMC11417281

[45] Porumb M, Stranges S, Pescapè A, Pecchia L (2020) Precision medicine and artificial intelligence: a pilot study on deep learning for hypoglycemic events detection based on ECG. Sci Rep 10(1):170. https://doi-org.proxy.bnl.lu/10.1038/s41598-019-56927-5 10.1038/s41598-019-56927-5 PMC6957484 31932608

[46] Katsarou DN, Georga EI, Sakaloglou P et al (2024) Glucose prediction using population-based models and genetic data in type 1 diabetes patients. Annu Int Conf IEEE Eng Med Biol Soc 2024:1–4. 10.1109/embc53108.2024.10782264 40039688 10.1109/EMBC53108.2024.10782264

