# Supplementary material for: Time below range alone is insufficient to identify severe hypoglycaemia risk in type 1 diabetes—the critical role of hypoglycaemia awareness: results from the SFDT1 study
Source: Diabetologia. 2025 Sep 9;68(12):2719–31. doi: 10.1007/s00125-025-06536-x (PMC12594687; doi:10.1007/s00125-025-06536-x)
Supplement: Supplementary file 1 — ESM (PDF 526 KB) [file 125_2025_6536_MOESM1_ESM.pdf]

ELECTRONIC SUPPLEMENTARY MATERIAL

Contents

Fig. 1. Timeline information of the questionnaires. Other individual, clinical and treatment variables were collected at baseline, during the inclusion visit ... 2

Fig. 2. Flowchart of study participants with type 1 diabetes from SFDT1 cohort ... 2

Fig. 3. Spline logistic regression analysis of the predicted probability (risk) of an SHE during the follow-up year based on TBR54 at baseline in individuals with no IAH vs with IAH. The plot displays the 95% CI of the predicted values ... 2

Table 1. Number (percentage) of missing values for included variables (N = 848). ... 3

Table 2. Comparison of included (filled ePRO 12M) and excluded (did not fill ePRO 12M) SFDT1 participants. ... 4

Table 3. Clinical phenotyping of SFDT1 cohort according to (baseline) TBR70 categories. ... 6

Table 4. Clinical phenotyping of SFDT1 cohort according to (baseline) TBR54 categories. ... 8

Principal Investigators - SFDT1 centres ... 10

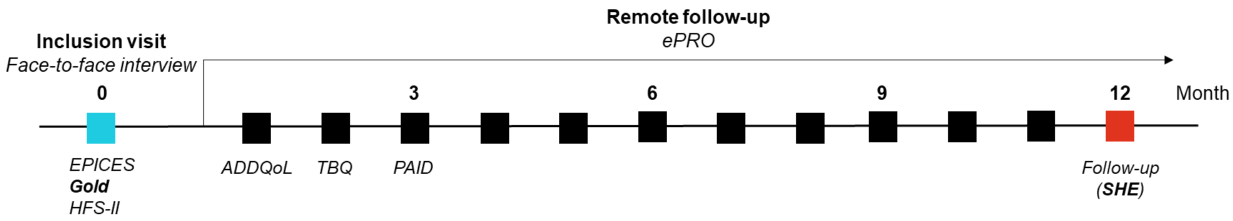

Fig. 1. Timeline information of the questionnaires. Other individual, clinical and treatment variables were collected at baseline, during the inclusion visit

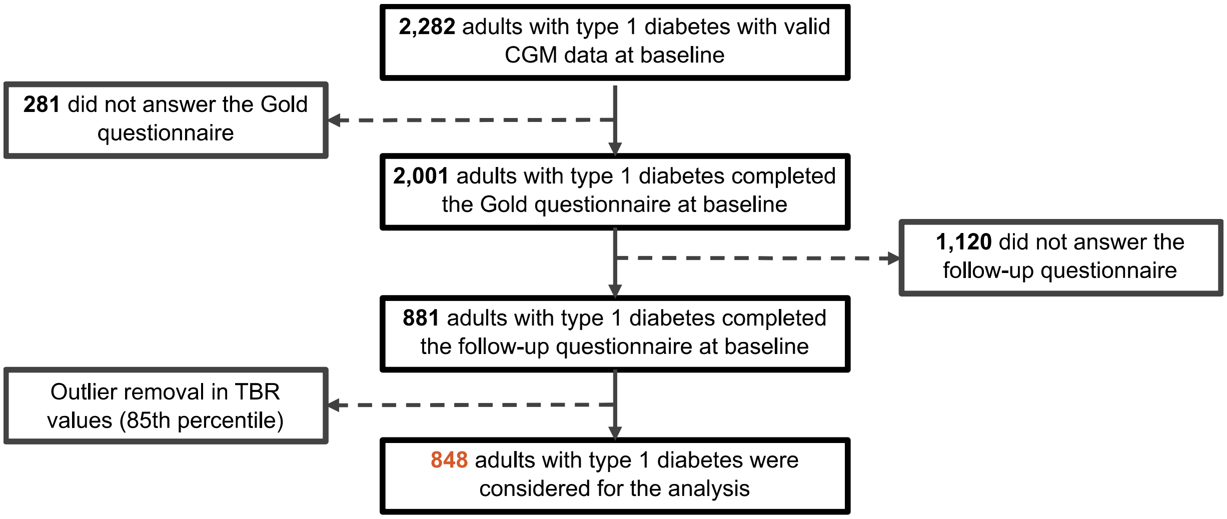

Fig. 2. Flowchart of study participants with type 1 diabetes from SFDT1 cohort

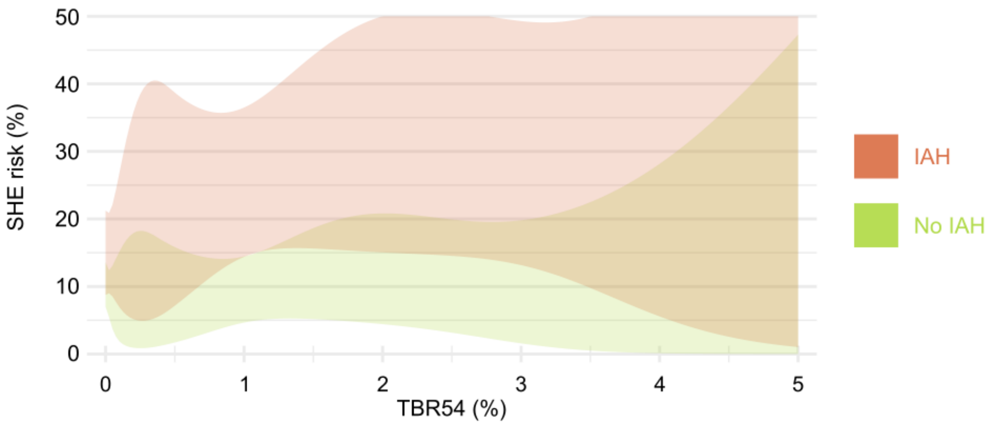

Fig. 3. Spline logistic regression analysis of the predicted probability (risk) of an SHE during the follow-up year based on TBR54 at baseline in individuals with no IAH vs with IAH. The plot displays the 95% CI of the predicted values

Table 1. Number (percentage) of missing values for included variables (N = 848).

Variable	Missing, N (%)	Variable	Missing, N (%)
**Individual**		**Complications**	
Age	0 (0.0)	Acute complications (at least one event during the previous year)	
Female	0 (0.0)	Severe hypoglycaemia	0 (0.0)
Diabetes duration	0 (0.0)	Diabetic ketoacidosis	0 (0.0)
Higher education	17 (2.0)	Long-term complications	
Socially vulnerable	72 (8.5)	CVD	1 (0.1)
Alcohol consumption	1 (0.1)	Retinopathy	1 (0.1)
Smoking status	1 (0.1)	Nephropathy	286 (33.7)
**Clinical**		Neuropathy	15 (1.8)
CGM brand	3 (0.4)	**Patient-reported outcomes**	
Insulin treatment	2 (0.2)	ADDQoL, average weighted impact	10 (1.2)
BMI	19 (2.2)	IAH status	0 (0.0)
HbA1c	8 (0.9)	Affective Fear of Hypoglycaemia	116 (13.7)
TBR54	0 (0.0)	Behavioural Avoidance of Hypoglycaemia	102 (12.0)
TBR70	0 (0.0)	Treatment burden	285 (33.6)
TIR	0 (0.0)	Diabetes distress PAID	29 (3.4)
TAR	0 (0.0)		
GRI	0 (0.0)		
Coefficient of Variation	128 (15.1)		
Systolic blood pressure	19 (2.2)		
Heart rate	19 (2.2)		
Total Cholesterol	96 (11.3)		
LDL-cholesterol	95 (11.2)		
HDL-cholesterol	92 (10.8)		
Triglycerides	93 (11.0)		

Table 2. Comparison of included (filled ePRO 12M) and excluded (did not fill ePRO 12M) SFDT1 participants.

Baseline characteristics	Included (N = 848)	Excluded (N = 1,079)	*p* value
**Individual**			
Age, years	41.60±13.31)	38.45±14.46)	<0.001
Female sex, *n* (%)	456 (53.8)	517 (47.9)	0.012
Diabetes duration, years	23.79±13.74)	20.67±13.98)	<0.001
Higher education, *n* (%)	609 (71.8)	635 (58.9)	<0.001
Socially vulnerable, *n* (%)	138 (16.3)	254 (23.5)	<0.001
Alcohol consumption, *n* (%)			<0.001
Never	212 (25.0)	366 (33.9)	
Yes, but not in excess	595 (70.2)	644 (59.7)	
Yes, in excess	41 (4.8)	65 (6.0)	
Smoking status, *n* (%)			0.001
Never	488 (57.5)	612 (56.7)	
Past smoker	228 (26.9)	231 (21.4)	
Current smoker	132 (15.6)	228 (21.1)	
**Clinical**			
CGM brand, *n* (%)			<0.001
Abbott	168 (19.8)	155 (14.4)	
Dexcom	108 (12.7)	179 (16.6)	
Medtronic	144 (17.0)	132 (12.2)	
Insulin treatment, *n* (%)			<0.001
MDI	257 (30.3)	450 (41.7)	
Only pump	182 (21.5)	141 (13.1)	
Open Loop	257 (30.3)	292 (27.1)	
AID	152 (17.9)	187 (17.3)	
BMI	25.51 (23.05–28.73)	24.91 (22.48–28.36)	0.003
HbA1c, mmol/mol	56.27 (50.81–62.83)	58.46 (50.81–66.11)	<0.001
HbA1c, %	7.30 (6.80–7.90)	7.50 (6.80–8.20)	
TBR54 <1%, *n* (%)	584 (68.9)	795 (73.7)	0.023
TBR70 (<70 mg/dl), %	3.00 (1.00–6.00)	2.80 (1.00–5.00)	0.069
TBR70 <4%, *n* (%)	501 (59.1)	668 (61.9)	0.224
TIR, %	59.99±15.66)	57.16±17.22)	<0.001
TAR (>180 mg/dl), %	36.12±16.22)	39.24±18.02)	<0.001
GRI, %	57.30±28.45)	63.37±33.72)	<0.001
CV, %	37.60 (33.90–41.00)	37.60 (33.50–41.90)	0.849
Systolic BP, mmHg	124.36±16.93)	123.53±16.30)	0.278

Heart rate, beats/min	74.97±13.55)	75.18±14.10)	0.736
Total Cholesterol, mmol/l	4.50 (3.90–5.07)	4.41 (3.80–5.02)	0.020
LDL, mmol/l	2.55±0.81)	2.49±0.84)	0.123
HDL, mmol/l	1.56 (1.32–1.81)	1.47 (1.19–1.77)	<0.001
Triglycerides, mmol/l	0.78 (0.63–1.01)	0.82 (0.62–1.10)	0.047
**Complications**			
Acute complications (at least one event during the previous year), *n* (%)			
Severe hypoglycaemia	68 (8.0)	101 (9.4)	0.341
Diabetic ketoacidosis	27 (3.2)	63 (5.8)	0.009
Long-term complications, *n* (%)			
CVD	41 (4.8)	32 (3.0)	0.048
Retinopathy	264 (31.1)	371 (34.4)	0.130
Nephropathy	210 (24.8)	215 (19.9)	<0.001
Neuropathy	305 (36.0)	328 (30.4)	0.033
**Patient-reported outcomes**			
ADDQoL, average weighted impact score [-9 to 3]	-2.55±1.66	-2.49±1.81	0.524
Hypoglycaemia awareness, *n* (%)			0.022
Aware	514 (60.6)	704 (65.2)	
Undetermined	135 (15.9)	127 (11.8)	
Impaired	199 (23.5)	248 (23.0)	
Affective fear of hypoglycaemia score [0–16]	4.81 (3.34)	4.81 (3.48)	0.989
Behavioural avoidance of hypoglycaemia score [0–16]	6.73±2.89	6.79±3.08	0.648
Treatment burden score [0–150]	60.21±28.42	59.27±31.05[a]	0.621
Diabetes distress PAID score [0–100]	39.65±20.41	39.63±21.17[a]	0.989

Data are shown as mean ±SD, median (IQR) or n (%); values within square brackets indicate minimum–maximum values for that variable

p values were calculated using t test or Wilcoxon test to compare group differences with continuous outcomes and $\chi 2$ test to compare categorical variables

[a] More than 50% missing data

Table 3. Clinical phenotyping of SFDT1 cohort according to (baseline) TBR70 categories.

Characteristics	TBR70 ≤1% (N=265)	1%< TBR70 <4% (N=236)	4%≤ TBR70 ≤6% (N=180)	TBR70 >6% (N=167)	*p* value
**Individual**					
Age, years	41.60±13.47	41.60±13.32	41.87±13.27	41.34±13.19	0.987
Female sex, *n* (%)	150 (56.6)	129 (54.7)	94 (52.2)	83 (49.7)	0.529
Diabetes duration, years	22.99±14.54	23.58±13.54	24.68±13.97	24.41±12.40	0.564
Higher education, *n* (%)	190 (71.7)	171 (72.5)	124 (68.9)	124 (74.3)	0.729
Socially vulnerable, *n* (%)	45 (17.0)	33 (14.0)	34 (18.9)	26 (15.6)	0.579
Alcohol consumption, *n* (%)					0.740
Never	74 (27.9)	56 (23.7)	42 (23.3)	40 (24.0)	
Yes, but not in excess	176 (66.4)	169 (71.6)	132 (73.3)	118 (70.7)	
Yes, in excess	15 (5.7)	11 (4.7)	6 (3.3)	9 (5.4)	
Smoking status, *n* (%)					0.397
Never	148 (55.8)	135 (57.2)	100 (55.6)	105 (62.9)	
Past smoker	81 (30.6)	62 (26.3)	51 (28.3)	34 (20.4)	
Current smoker	36 (13.6)	39 (16.5)	29 (16.1)	28 (16.8)	
**Clinical**					
CGM brand, *n* (%)					<0.001
Abbott	149 (56.2)	159 (67.4)	137 (76.1)	151 (90.4)	
Dexcom	43 (16.2)	44 (18.6)	15 (8.3)	6 (3.6)	
Medtronic	73 (27.5)	33 (14.0)	28 (15.6)	10 (6.0)	
Insulin treatment, *n* (%)					<0.001
MDI	77 (29.1)	57 (24.2)	54 (30.0)	69 (41.3)	
Only pump	49 (18.5)	57 (24.2)	39 (21.7)	37 (22.2)	
Open Loop	64 (24.2)	78 (33.1)	62 (34.4)	53 (31.7)	
AID	75 (28.3)	44 (18.6)	25 (13.9)	8 (4.8)	
BMI	26.12 (23.34–30.09)	25.27 (23.26–27.99)	25.58 (23.44–28.82)	24.93 (22.51–28.41)	0.032
HbA1c, mmol/mol	58.46 (51.90–65.02)	56.27 (50.67–62.83)	55.18 (50.54–60.65)	54.09 (49.72–60.65)	<0.001
HbA1c, %	7.50 (6.90–8.10)	7.30 (6.79–7.90)	7.20 (6.77–7.70)	7.10 (6.70–7.70)	
TBR54 (<54 mg/dl), %	0.00 (0.00–0.00)	0.00 (0.00–0.50)	0.85 (0.00–1.00)	2.00 (0.00–3.00)	<0.001
TBR70 (<70 mg/dl), %	1.00 (0.00–1.00)	2.50 (2.00–3.00)	5.00 (4.00–6.00)	9.00 (7.65–11.00)	<0.001
TIR, %	57.86±19.29	60.74±14.89	61.83±12.96	60.35±12.36	0.045
TAR (>180 mg/dl), %	41.50±19.40	36.77±14.91	33.27±12.96	29.73±12.30	<0.001
GRI, %	55.97±35.41	54.09±26.59	55.20±22.88	66.22±21.65	<0.001
CV, %	33.80 (30.30–37.37)	37.00 (34.15–40.02)	39.30 (36.68–42.20)	42.60 (39.15–46.65)	<0.001
Systolic BP, mmHg	125.39±17.16	122.72±17.58	125.00±17.05	124.36±15.44	0.328

Heart rate, beats/min	75.80±13.27	75.12±13.49	73.68±13.59	74.81±14.07	0.442
Total Cholesterol, mmol/l	4.57 (3.90–5.15)	4.58 (4.08–5.15)	4.40 (3.88–5.01)	4.41 (3.80–4.89)	0.102
LDL, mmol/l	2.61±0.83	2.55±0.76	2.55±0.81	2.48±0.84	0.484
HDL, mmol/l	1.57 (1.27–1.81)	1.60 (1.39–1.84)	1.53 (1.32–1.80)	1.53 (1.32–1.82)	0.229
Triglycerides, mmol/l	0.81 (0.67–1.08)	0.76 (0.61–0.97)	0.80 (0.63–1.01)	0.74 (0.61–0.95)	0.010
**Complications**					
Acute complications (at least one event during the previous year), *n* (%)					
Severe hypoglycaemia	21 (7.9)	13 (5.5)	13 (7.2)	21 (12.6)	0.076
Diabetic ketoacidosis	9 (3.4)	10 (4.2)	7 (3.9)	1 (0.6)	0.187
Incident SHE, *n* (%) (during 1-year follow-up)	32 (12.1)	24 (10.2)	19 (10.6)	24 (14.4)	0.582
Long-term complications, *n* (%)					
CVD	12 (4.5)	14 (5.9)	9 (5.0)	6 (3.6)	0.743
Retinopathy	86 (32.5)	69 (29.2)	61 (33.9)	48 (28.7)	0.638
Nephropathy	64 (24.2)	55 (23.3)	52 (28.9)	39 (23.4)	0.543
Neuropathy	87 (32.8)	92 (39.0)	65 (36.1)	61 (36.5)	0.554
**Patient-reported outcomes**					
ADDQoL, average weighted impact score [-9 to 3]	-2.54±1.68	-2.57±1.64	-2.38±1.55	-2.70±1.77	0.330
Gold Score	2.00 (2.00–3.00)	2.00 (1.00–3.00)	2.00 (1.00–3.00)	2.00 (2.00–4.00)	0.003
Hypoglycaemia awareness, *n* (%)					0.023
Aware	155 (58.5)	162 (68.6)	112 (62.2)	85 (50.9)	
Undetermined	47 (17.7)	28 (11.9)	25 (13.9)	35 (21.0)	
Impaired	63 (23.8)	46 (19.5)	43 (23.9)	47 (28.1)	
Affective fear of hypoglycaemia score [0–16]	5.06±3.39	4.59±3.25	4.46±3.46	5.11±3.22	0.113
Behavioural avoidance of hypoglycaemia score [0–16]	7.09±3.03	6.64±2.86	6.51±2.82	6.53±2.76	0.104
Treatment burden score [0–150]	62.57±28.07	59.57±28.09	57.86±29.88	59.90±27.83	0.363
Diabetes distress PAID score [0–100]	40.76±20.64	38.52±20.35	38.44±20.90	40.79±19.62	0.447

Data are shown as mean ±SD, median (IQR) or *n* (%); values within square brackets indicate minimum–maximum values for that variable

p values were calculated using *t* test or Wilcoxon test to compare group differences with continuous outcomes and $\chi 2$ test to compare categorical variables

Table 4. Clinical phenotyping of SFDT1 cohort according to (baseline) TBR54 categories.

Characteristics	TBR54 <1% (N=584)	TBR54 ≥1% (N=264)	*p* value
**Individual**			
Age, years	42.30±13.70	40.07±12.28	0.024
Female sex, *n* (%)	318 (54.5)	138 (52.3)	0.607
Diabetes duration, years	24.14±14.19	23.02±12.66	0.271
Higher education, *n* (%)	427 (73.1)	182 (68.9)	0.242
Socially vulnerable, *n* (%)	485 (83.0)	225 (85.2)	0.487
Alcohol consumption, *n* (%)			0.693
Never	151 (25.9)	61 (23.1)	
Yes, but not in excess	405 (69.3)	190 (72.0)	
Yes, in excess	28 (4.8)	13 (4.9)	
Smoking status, *n* (%)			0.005
Never	330 (56.5)	158 (59.8)	
Past smoker	174 (29.8)	54 (20.5)	
Current smoker	80 (13.7)	52 (19.7)	
**Clinical**			
CGM brand, *n* (%)			0.086
Abbott	398 (68.2)	198 (75.0)	
Dexcom	83 (14.2)	25 (9.5)	
Medtronic	103 (17.6)	41 (15.5)	
Insulin treatment, *n* (%)			0.242
MDI	169 (28.9)	88 (33.3)	
Only pump	122 (20.9)	60 (22.7)	
Open Loop	179 (30.7)	78 (29.5)	
AID	114 (19.5)	38 (14.4)	
BMI	25.79 (23.39–28.96)	24.68 (22.53–28.40)	0.005
HbA1c, mmol/mol	56.27 (50.81–62.83)	55.73 (50.81–62.83)	0.776
HbA1c, %	7.30 (6.80–7.90)	7.25 (6.80–7.90)	
TBR54 (<54 mg/dl), %	0.00 (0.00–0.00)	1.00 (1.00–2.00)	<0.001
TBR70 (<70 mg/dl), %	2.00 (1.00–3.00)	6.00 (4.00–9.00)	<0.001
TIR, %	59.81±16.92	60.40±12.45	0.608
TAR (>180 mg/dl), %	37.59±17.33	32.86±12.87	<0.001
GRI, %	53.76±29.58	65.14±24.04	<0.001
CV, %	36.30 (32.68–39.54)	40.45 (37.27–44.80)	<0.001

Systolic BP, mmHg	124.73±17.67	123.55±15.18	0.349
Heart rate, beats/min	74.83±13.13	75.28±14.48	0.651
Total Cholesterol, mmol/l	4.55 (3.95–5.10)	4.42 (3.82–4.92)	0.104
LDL, mmol/l	2.56±0.79	2.53±0.85	0.641
HDL, mmol/l	1.58 (1.32–1.85)	1.50 (1.30–1.77)	0.047
Triglycerides, mmol/l	0.79 (0.64–1.03)	0.76 (0.62–0.98)	0.068
**Complications**			
Acute complications (at least one event during the previous year), *n* (%)			
Severe hypoglycaemia	41 (7.0)	27 (10.2)	0.146
Diabetic ketoacidosis	64 (11.0)	35 (13.3)	0.396
Incident SHE, *n* (%) (during 1-year follow-up)	23 (3.9)	4 (1.5)	0.099
Long-term complications, *n* (%)			
CVD	31 (5.3)	10 (3.8)	0.434
Retinopathy	185 (31.7)	79 (29.9)	0.667
Nephropathy	139 (23.8)	71 (26.9)	0.379
Neuropathy	203 (34.8)	102 (38.6)	0.312
**Patient-reported outcomes**			
ADDQoL, average weighted impact score [-9 to 3]	-2.52±1.66	-2.61±1.66	0.430
Gold Score	2.00 (1.00–3.00)	2.00 (1.00–3.00)	0.809
Hypoglycaemia awareness, *n* (%)			0.921
Aware	355 (60.8)	159 (60.2)	
Undetermined	91 (15.6)	44 (16.7)	
Impaired	138 (23.6)	61 (23.1)	
Affective fear of hypoglycaemia score [0–16]	4.71±3.30	5.04±3.42	0.187
Behavioural avoidance of hypoglycaemia score [0–16]	6.71±2.94	6.77±2.79	0.806
Treatment burden score [0–150]	60.67±28.22	59.19±28.89	0.485
Diabetes distress PAID score [0–100]	39.69±20.78	39.57±19.61	0.939

Data are shown as mean ±SD, median (IQR) or *n* (%); values within square brackets indicate minimum–maximum values for that variable

p values were calculated using *t* test or Wilcoxon test to compare group differences with continuous outcomes and $\chi 2$ test to compare categorical variables

Principal Investigators - SFDT1 centres

001 - Lariboisière, Pr Jean-Pierre Riveline, Dr Jean-Baptiste Julla, Dr Tiphaine Vidal-Trécan, Dr Vanessa Juddoo

002 - Dijon, Pr Bruno Vergès

003 - Le Creusot, Dr Sylvaine Clavel, Dr Hermione Agopian

004 - Avicenne, Pr Emmanuel Cosson, Dr Sopio Tatulashvili

005 - Cabinet Libéral Dijon, Dr Picard

006 - Guadeloupe, Pr Fritz-Line Velayoudom, Dr Alexandra Prevot, Dr Claude Gruel, Dr Ingrid Cirederf

007 - Diab-Ecare Lyon, Pr Charles Thivolet, Dr Mélanie Gaudilliere, Dr Sylvie Villar-Fimbel, Dr Béatrice Mestre Filippini

008 - Robert Debré, Paris, Dr Elise Bismuth, Dr Hassina Benlarbi

009 - Necker, Paris, Pr Jacques Beltrand, Dr Alix Besançon

010 - Grenoble, Pr Pierre-Yves Benhamou, Pr Sandrine Lablanche, Dr Manon Jalbert

011 - Bichat, Paris, Dr Louis Potier, Dr Aurélie Carlier

012 - Cochin, Paris, Dr Agnès Sola

013 - Strasbourg, Pr Nathalie Jeandidier, Pr Laurence Kessler, Dr Marion Munch

014 - Toulouse, Pr Hélène Hanaire, Pr Pierre Gourdy, Dr Raquel Tirado, Dr Vincent Melki

015 - Jean Verdier, Dr Sarah Pinto

016 - Caen, Pr Yves Reznik, Dr Bleuenn Dreves, Pr Michael Joubert, Dr Anne Rod

017 - Besançon, Dr Sophie Borot, Dr Annie Clergeot

018 - Diab-Ecare Lyon (Pédiatrie), Dr Nathalie Bendelac

019 - Bordeaux, Pr Vincent Rigalleau, Dr Amandine Ferriere, Dr Alice Larroumet

020 - Rennes, Dr Isabelle Guilhem, Dr Christele Derrien, Dr Agathe Guenego, Dr Patricia Vaduva, Dr Elsa Bleichner, Dr Emeric Rageul, Dr Margot De La Vergne De Cerval, Dr Annabelle Esvant, Dr Mathilde Fichet, Dr Audrey Durous

021 - Cemm Strasbourg, Dr Laurent Meyer

022 - Bégin, Pr Lyse Bordier, Dr Cyril Garcia, Dr Sika Nassouri, Dr Mathilde Sollier

023 - Nancy, Pr Bruno Guerci, Dr Stéphanie Jellimann, Dr Philip Böhme, Dr Nicolas Scheyer, Dr Mazen Soliman, Dr Meliha Mahmutovic, Dr Laura Mayer

024 - Sud Francilien, Dr Sylvia Franc, Pr Alfred Penfornis, Dr Coralie Amadou

025 - Nice, Pr Nicolas Chevalier, Dr Anne-Gaëlle Decoux-Poullot, Dr Stéphanie Palle Defille

026 - Rouen, Pr Gaetan Prevost, Dr Lucile Moreau Grange, Dr Karine Durand, Dr Florence Eas

027 - Montpellier, Pr Eric Renard, Dr Anne Farret, Dr Florence Galtier

028 - Reims, Pr Brigitte Delemer

029 - Hegp, Pr Claire Carette, Dr Alina Radu, Dr Amel Ait Boudaoud Hansal

030 - Nantes, Pr Samy Hadjadj, Dr Pierre Morcel

032 - Jean Verdier (Pédiatrie), Dr Nadine Lucidarme

033 - Clermont Ferrand, Pr Igor Tauveron, Dr Thomas Benichou, Dr Sylvain Roumeau, Sarah Dallel, Dr Mathilde Picard

034 - Reims (Pédiatrie), Dr Pierre-François Souchon, Dr Anne Sophie Salmon - Musial, Dr Aurelie Berot

035 - Saint Joseph St Luc, Dr Lucien Marchand, Dr Cédric Luyton

037 - St Joseph (Paris), Dr Isabela Banu, Dr Olivier Dupuy, Dr Adèle Voican

038 - Aix-En-Provence, Dr Blandine Delenne, Dr Flavia Bazin

039 - Lille, Pr Anne Vambergue, Dr Florence Baudoux

040 - La Rochelle, Dr Didier Gouet

041 - Brest, Dr Emmanuel Sonnet, Dr Nathalie Roudaut, Dr Philippe Thuillier

042 - HCL Lyon Sud, Pr Emmanuel Disse, Dr Amal Lemoine

043 - Brest (Pédiatrie), Dr Karine Bourdet, Dr Béatrice Gueguen

044 - Grenoble (Pédiatrie), Dr Anne Spiteri, Dr Justine Suler, Dr Cyril Ruello

045 - Nantes (Pédiatrie), Dr Sabine Baron, Dr Vanessa Menut

047 - Marseille La conception, Dr Valero, Pr Sophie Béliard, Dr Noémie Dubois, Dr Rachel Grangeot, Dr Florian Mourre, Dr Audrey Bégu Le Corroller

048 - Tours, Pr Pierre Henri Ducluzeau, Dr Lise Crinière, Dr Hélène Champion, Dr Peggy Renoult Pierre

049 - Compiègne, Dr Kalliopi Bilariki, Dr Etienne Justinien, Dr Sarra El Younsi-Hammani, Dr Jean Noël Barjon

050 - Vannes, Dr Gwenaelle Arnault, Dr Mathieu Giraud

051 - Marseille (Pédiatrie), Pr Rachel Reynaud, Dr Delphine Bernoux

053 - Cabinet Libéral Sokol, Dr Emmanuelle Sokol

054 - Saint Antoine, Dr Camille Vatier

055 - Agen Nerac, Dr Bénédicte Fremy, Dr Florent Verdier

057 - Rennes (Pédiatrie), Dr Marie Béatrice Saade, Dr Sylvie Nivot, Dr Magali Cornier, Dr Marie Aline Guitteny, Dr Emmanuelle Nivet

058 - Besançon (Pédiatrie), Dr Mignot, Dr Maïté Gebhart, Dr Claire Ballot Schmit, Dr Claire Sechter

059 - Limoges, Dr Laurence Salle, Dr Sophie Galinat

060 - Cabinet Libéral Dr Sanz, Dr Caroline Sanz

061 - Strasbourg (Pédiatrie), Dr Marie Mansilla, Dr Sylvie Rossignol

062 - CH Gonesse, Dr Jennifer Allain

063 - CH Simone Veil, Dr Diane-Cécile Gauthier, Dr Karim Lachgar

064 - Hôpital St Joseph Marseille, Dr Lise Dufaitre Patouraux, Dr Julien Negre

065 - Hôpital Nord Marseille, Dr Olivia Ronsin

066 - Cabinet libéral Dr Ronci, Dr Nathalie Ronci

067 - CH Saint-Nazaire (pédiatrie), Dr Marie Dupré, Dr Sandie Jore

068 - Hôpital européen Marseille, Dr Pauline Schaepelynck

069 - CH Vienne, Dr Amal Lemoine

070 - CHU Angers, Dr Claire Briet

071 - CHU Dijon (pédiatrie), Dr Candace Ben Signor

072 - CH Béthune, Dr Christine Lemaire

073 - CHU Rouen (pédiatrie), Dr Alexandre Naccache, Dr Mireille Castanet, Dr Claire Gayet

074 - CH Saint Brieuc, Dr Oana Ionesco

075 - Hôpital NOVO, Dr Catherine Campinos Imberti

076 - CH Boulogne sur mer, Dr Marie Le Page, Dr Thomas Lemoine, Dr Agathe Poussin, Dr Camille Duhamel

077 - CHU Orléans, Dr Elise Mongeois, Dr Elise Pinto Da Rocha, Dr Lucie Cloix, Dr Grégoire Fauchier, Dr Elena-Loredana Simionescu, Dr Sarah Foldil-Cherif

078 - CH Le Havre, Dr Déborah Ancelle

079 - CH Dunkerque (pédiatrie), Dr Harmonie Mazoyer
